# Microcalcification Segmentation from Mammograms: A Morphological Approach

**DOI:** 10.1007/s10278-016-9923-8

**Published:** 2016-11-14

**Authors:** Marcin Ciecholewski

**Affiliations:** Faculty of Mathematics and Computer Science, Jagiellonian University, ul. Łojasiewicza 6, 30-348 Kraków, Poland

**Keywords:** Mathematical morphology, Image processing, Segmentation, Microcalcification, Breast cancer, Mammography

## Abstract

This publication presents a computer method for segmenting microcalcifications in mammograms. It makes use of morphological transformations and is composed of two parts. The first part detects microcalcifications morphologically, thus allowing the approximate area of their occurrence to be determined, the contrast to be improved, and noise to be reduced in the mammograms. In the second part, a watershed segmentation of microcalcifications is carried out. This study was carried out on a test set containing 200 ROIs 512 × 512 pixels in size, taken from mammograms from the Digital Database for Screening Mammography (DDSM), including 100 cases showing malignant lesions and 100 cases showing benign ones. The experiments carried out yielded the following average values of the measured indices: 80.5% (similarity index), 75.7% (overlap fraction), 70.8% (overlap value), and 19.8% (extra fraction). The average time of executing all steps of the methods used for a single ROI amounted to 0.83 s.

## Introduction

**Fig. 1:**
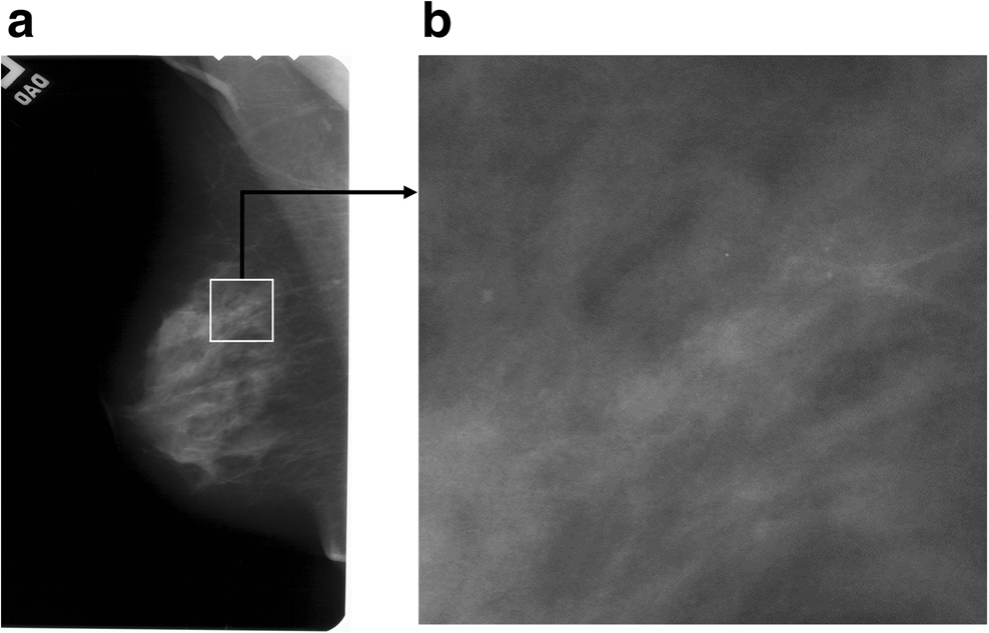
Cranio-caudal view of an example mammogram A_1553_1.LEFT_CC. **a** Mammogram A_1553_1.LEFT_CC with a white rectangular ROI marked. **b** Enlarged ROI 512 × 512 pixels in size extracted from item (**a**)

**Fig. 2:**
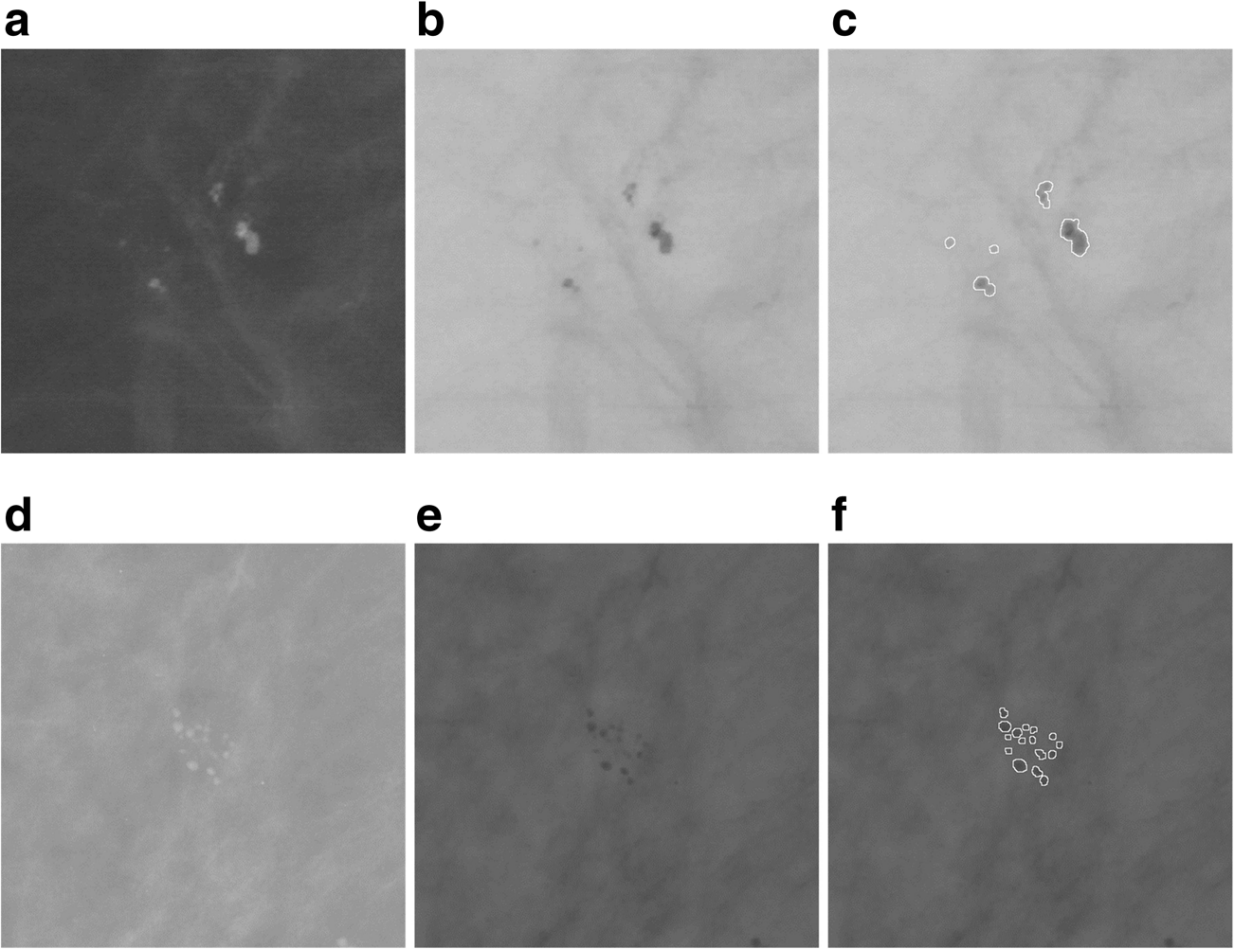
Two examples of patches with microcalcifications: benign (upper row, based on the image A_1551_1.LEFT_MLO), malignant (lower row, based on the image A_1214_LEFT_MLO). *First column*: mammographic image patches. *Second column*: the image inverted in gray levels. *Third column*: segmentation—all microcalcifications have been marked

In mammography imaging, the presence of microcalcifications, i.e., small deposits of calcium in the breast, is the primary indicator of breast cancer. However, not all microcalcifications are proof of malignancy and their distribution within the breast can be used to determine whether the clusters contain benign lesions or constitute a threat indicating a malignancy. Microcalcifications presented in Figs. 1a and 2a and d are small deposits of calcium in the breast, which appear as small bright spots on mammograms. Unfortunately, the correct detection of microcalcifications in mammograms can often be very difficult. Breasts contain variable quantities of glandular, fatty, and connective tissues, and if there are a lot of glandular tissues, the mammograms are very bright, which makes small microcalcifications poorly visible [1]. If a physician has to examine numerous series of mammograms, their visual assessment capacity is greatly reduced. Consequently, computer-aided diagnosis (CAD) is being developed to make the diagnostic process easier for the radiologists [2–7]. The standard functions of CAD systems comprise the segmentation [8–11], feature extraction [12–15], and classification [5, 16–19] to determine whether lesions are present.

Although the improvement of each of the listed functions raises the capacity of the system, the segmentation can be considered the most significant, as the precise segmentation of lesions impacts the extraction of features and the classification. Microcalcifications were segmented using several techniques, such as morphological filters [1, 20–23], machine learning [11, 24], and the wavelet transform [25] method using normalized Tsallis Entropy and fuzzy sets [10]. Most recent research based on machine learning [24], the wavelet transform [25], and active contour [8, 9] demonstrate that microcalcification segmentation is highly significant and the researchers report good results of the methods proposed.

Chen et al. [24] analyzed the topology/connectivity of individual microcalcifications inside a cluster using multiscale morphology. In [24], microcalcifications were segmented using a knowledge-based approach [11] with the application of machine learning methods like the pixel-based boosting classifier which automatically allows the most salient features of microcalcifications to be selected. Chen et al. [24] report high classification accuracies (up to 96%) and also good ROC (region of convergence) results achieved.

Batchelder [25] proposed the 2D wavelet transform modulus maxima method (WTMM) to detect microcalcifications in mammograms. Then, fractal geometry was used to determine benign and malignant microcalcification clusters, and in particular, a “fractal zone” and “Euclidean zones” (non-fractal) were defined. The authors analyzed 118 images of 59 patients. According to their results, the probability that fractal breast lesions are malignant is between 74 and 98% and the probability that Euclidean breast lesions are benign is between 76 and 96%.

Arikidis et al. [8] presented multiscale active contours method (MAC) which enable single microcalcifications to be segmented. This method requires the seed contour to be initiated manually. In [8], rectangular ROIs 81 × 81 pixels in size were analyzed and experiments were carried out for the DDSM database, with the reported mean value of the area overlap measure of 0.61 ± 0.15.

Duarte et al. [9] presented a geometric active contour method (GAC) for segmenting single microcalcifications. In every instance, the active contour is initiated for a single microcalcification. In [9], researches used 1000 rectangular ROIs taken from mammograms from the DDSM database, sized from 20 × 20 pixels to 41 × 41 pixels. Duarte et al. [9] report that they obtained a mean value of the area overlap measure of 0.52 ± 0.20.

The purpose of this publication was to propose solutions for the following:

- Obtain the best possible segmentation results.
- Achieving a fast operation of all the methods used.

This project uses morphological image transformations [23, 26] to detect microcalcifications, and then watershed segmentation [23, 26] which makes it possible to extract the shape of microcalcifications just as in [22, 27]. In publications by Nieniewski [22, 27], user interaction is necessary to indicate the seed point of the watershed by immersion segmentation [28, 29]. In this project, the whole segmentation process is automated and does not require combining regions by maximizing average contrast, as was done in publications by Nieniewski [22, 27, 30]. This study makes use of other gradient transformations of the image undergoing watershed segmentation and fewer interim steps during the extraction of the final shape of microcalcifications. This makes it possible to execute the entire segmentation process in the mean time of 0.83 s.

## Materials and Methods

### Image Dataset Used

The research project used 220 ROIs with the constant dimensions of 512 × 512 pixels, in an 8-bit format, obtained from mammograms with the original high resolution (43.5 and 50 μm/pixel, digitized using the following scanners—Howtek 960, Lumisys 200 Laser, and Howtek MultiRad850), which came from the publicly accessible DDSM database [31, 32]. Of that number, 110 ROIs contain benign lesions, and the remaining 110 ROIs show malignant cases. The images were selected by a breast radiologist with 10 years of experience and are mainly fatty breast cases from different patients. Each ROI corresponds to a different patient. It should be noted that 20 ROIs, or more exactly 10 benign and 10 malignant ones, were used to determine the necessary parameters allowing the segmentation process to be controlled. The remaining 200 ROIs were used to test the presented segmentation method, and the results obtained are presented in the experimental part of this article. Methods which can automatically mark suspicious-looking anomalies containing potential microcalcifications form a useful functionality of CAD software. Examples of their solutions can be found in literature [33, 34]. However, these methods might mark false-positive regions which contain no microcalcifications. Consequently, the correct identification of microcalcification regions by an experienced breast radiologist is indispensable in analyzing the disease. Solutions presented in this publication concern microcalcification segmentation and make use of rectangular ROIs marked by a radiologist on the source mammogram, with suspicious-looking anomalies located in their centers. This is illustrated in Fig. 1. For every case in the DDSM database, there is a radiological diagnosis available. In addition, for images with microcalcifications, a coded contour identifying the area in which microcalcifications occur called a ground truth area (GTA) is available. Each case has four images acquired in the CC and MLO projections for the left and the right breast. CC is the cranio-caudal projection showing that central and medial part of the mamma. MLO is the medio-lateral oblique projection. In the experiments, a single view was taken, namely the CC or MLO view for each patient.

### Detecting and Segmenting Microcalcifications in Mammograms

The computer-aided detection and segmentation of microcalcifications from mammograms is a complex process, also because these microcalcifications are often much dispersed in the analyzed images, have low contrast, and are difficult to distinguish from their surroundings. These features may make it difficult to correctly segment them. Brief characteristics of microcalcifications taken from [35] are presented below:

- Microcalcifications are small, from 0.1 to 1.0 mm. Their average size is 0.3 mm. Microcalcifications smaller than of 0.1 mm also occur and are often impossible to distinguish from high-frequency noise.
- Microcalcifications can differ in their shape, size, and the distribution within the mammary gland.
- They are characterized by a low contrast in mammograms.
- Sometimes they adhere closely to the tissues surrounding them.

In the light of the above difficulties, the method presented in this publication comprises two consecutive parts making use of the morphological processing of digital images [22, 26, 27], namely

- Morphologically detecting microcalcifications.
- Watershed segmentation of microcalcifications.

Detecting microcalcifications allows the contrast to be increased in the image, the noise to be removed from it, and also some of the false-positive signals of microcalcifications to be removed. The results obtained are treated as a “map” on which the approximate areas in which microcalcifications occur are marked and will be used as an auxiliary image for a more precise determination of their shape. The next step in working with mammograms is to segment microcalcifications. This will be done using the watershed segmentation [22, 26, 27] to more accurately extract microcalcification shapes. Knowing the shape of microcalcification is very important as, together with their other features, it can prove tumor malignancy. The following description can be given based on the recently published work by Chen et al. [24]:

- Malignant microcalcifications appear to be small, numerous (>5 concentrated on an area of 1 cm^2^), and distributed densely because they lie inside milk ducts and associated structures in the breast.
- Benign microcalcifications are generally larger, less numerous (<4–5 per 1 cm^2^), and more spread out because they form in the breast stroma, cysts, or benign masses.

These differences in the variability of the distribution, the size, and the number of microcalcifications in the ROIs analyzed allow radiologists to decide on the further assessment and the possible biopsy of the breast. Consequently, a correctly performed microcalcification segmentation can greatly simplify decision-taking for the doctors. Figure 2a–c shows example ROIs with benign microcalcifications. These microcalcifications are rather spread out, there are cases of a relatively larger size, and they are less numerous than malignant microcalcifications shown in Fig. 2d–f, which, in contrast, are small, numerous, and densely distributed.

### Morphological Detection of Microcalcification

**Fig. 3:**
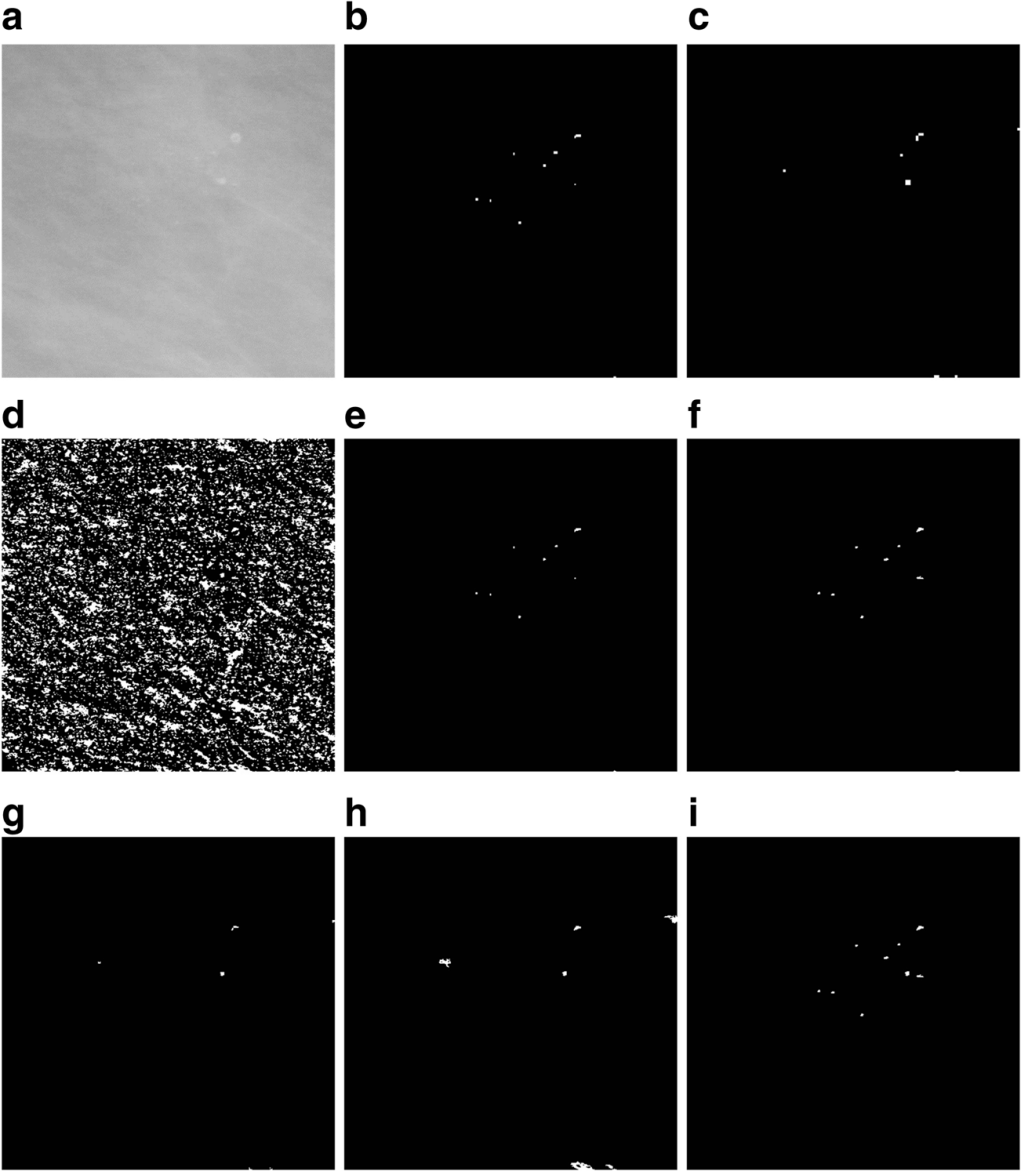
An illustration of detecting microcalcifications in an image 512 × 512 pixels in size, extracted from mammogram A_1131_1.RIGHT_MLO. **a** The result of microcalcifications detection in stage 1. **b**, **c** The result of using the detector based on Eq. (1) at the second and third levels of the morphological pyramid, respectively. **d** Extended maximum *emax*. **e** An image showing the marker for reconstructing microcalcifications detected at the second level of the morphological pyramid—an intersection of images from items (**b**) and (**d**). **f** The result of the reconstruction by dilation of the mask presented in item (**d**) and the marker presented in item (**e**), i.e., an image presenting microcalcifications detected at the second level of the morphological pyramid. **g** An image showing the marker for reconstructing microcalcifications detected at the third level of the morphological pyramid—an intersection of the images from items (**c**) and (**d**). **h** The result of the reconstruction by dilation of the mask presented in item (**d**) and the marker presented in item (**g**), i.e., an image presenting microcalcifications detected at the third level of the morphological pyramid. **i** The sum of images from items (**f**) and (**h**), using the OR operator, as the result of extracting microcalcifications using the morphological pyramid. The image has been subjected to additional “cleaning” operations

To detect microcalcifications, four stages of subsequent mammogram transformations are executed (shown in Fig. 3):

- Stage 1. The input mammogram marked *I* should be subjected to an operation of shifting it 21 gray levels down and then the same number of gray levels up in order to remove small brighter points in the darkest parts of the image, which could be wrongly recognized as microcalcifications. As a result of these operations, the variance of the image for gray levels between 0 and 21 will be removed. The output image is marked as *I*_2_.
- Stage 2. The second stage is about detecting microcalcifications of various sizes using the morphological pyramid and a structural element of the constant size of 3 × 3 pixels. The first level of the pyramid is the source image. The second level is obtained by applying first the closing-opening (C-O) filtration [26] with the aforementioned structural element to the source image, and then sampling every second pixel from the image. The third level is produced by conducting the same operations on the second level image. The C-O filtration and sampling produces an image size reduced twice but with the useful information about its objects retained. Microcalcifications are detected at the second and third levels of the pyramid, using the following formula:
  1 $$ T=I- min\left\{{\gamma}_S\left[{\varphi}_S(I)\right],I\right\} $$
  where *I* is the input image, *T* is the output image, *S* is a square structural element 3 × 3 pixels in size, *γ*_*S*_ and *φ*_*S*_ are, respectively, the opening and closing operations [26], and *min* represents the point minimum. The operation (1) detects small brighter parts of the image (Fig. 3b, c). Pixels with a less irregular brightness distribution in their surroundings receive a higher value. This transformation also constitutes de-noising filter. The *min* operation ensures that the result will never be negative.
  After the microcalcification detection at the second and third levels of the pyramid, the results are subjected to thresholding with the threshold equal to 4, i.e., pixels with their gray level below the threshold are assigned the value of 0 and the pixels with a gray level equal to 4 or higher are assigned the value of 255. If the threshold value was set lower, e.g., at 3, this produced too many potential microcalcification signals. The results of thresholding at the second and third level of the pyramid should then be reduced to the dimensions of the input image and sum up using the logical OR operator. The size of the input image can be restored by replacing every pixel with a block sized 2 × 2 for the second or 4 × 4 pixels for the third the pyramid level.
- Stage 3. The third stage consists in extracting all the brighter areas found in the *I*_2_ image produced in stage 1. This will be done by the morphological operation of the extended maximum *emax* [26], where image *I*_2_ is the mask and the image *I*_2_ after the number 5 is subtracted from all of its pixels is the marker:
  2 $$ {I}_{emax}={T}_1\left[h- convexit{y}_5\left({I}_2\right)\right] $$
  The *I*_*emax*_ image is composed of both microcalcifications and other brighter areas of image *I*_2_ (Fig. 3d). In the experiments forming part of this project, the value of *h* was adopted as 5 because higher values made the bright area too large. However, in some cases, the image will contain excessively large areas that do not correspond to the physical dimensions of microcalcifications. Such areas must be removed from the image as part of a separate operation. It was decided to eliminate potential signals of microcalcifications inside which a vertical, horizontal, left diagonal, or right diagonal chord 50 pixels or less in length can be drawn. These objects are deleted using erosion carried out separately for every one of four linear structural elements lying along the above directions. Erosion results should be summed up logically. The summed up erosion results will serve as a marker for the reconstruction by dilating large regions from the *I*_*emax*_ image. The *I*_*emax*_ image will be the mask in this reconstruction. The result of calculations at this stage is the difference between the *I*_*emax*_ image and the image produced by the reconstruction.
- Stage 4. The purpose of the last stage is to extract the area occupied by microcalcifications detected using Eq. (2). This will consist in reconstructing the appropriate areas of image *I*_*emax*_ indicated by signals detected at the second and third levels of the morphological pyramid. The logical overlap of the image indicating the microcalcifications at the given level of the pyramid and the *I*_*emax*_ image will constitute the marker in the reconstruction. The reconstruction results from the second and third levels of the pyramid should then be summed up using the logical OR operator, and as a result the so-called microcalcification “map” should be produced (Fig. 3i).

In addition, the image obtained at stage 4 was subjected to “cleaning” operations, namely

- Removing potential microcalcification areas located close to the edge of the image using the reconstruction.
- Removing potential microcalcification areas smaller than 10 pixels and larger than 70 pixels in area by area opening [26] and the logical subtraction of the images.
- Closing holes in image objects.

### The Watershed Segmentation of Microcalcifications

**Fig. 4:**
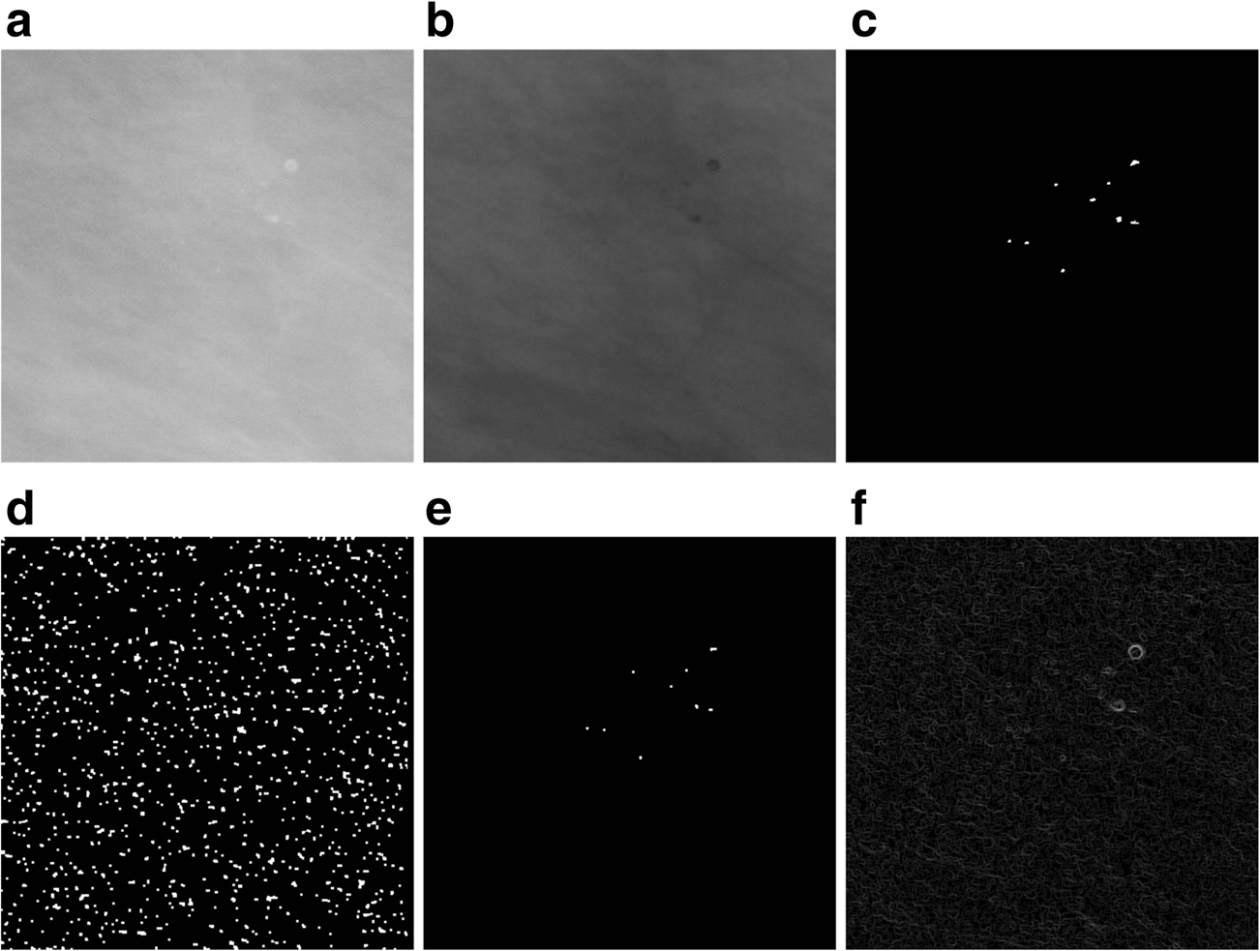
An illustration of extracting the microcalcifications shape. Part 1. **a** Image A_1131_1.RIGHT. **b** The filtered and inverted image from item (**a**). **c** The result of extracting microcalcifications using the morphological pyramid from Fig. 3. **d** Regional minima obtained for the image from item (**b**). **e** The intersection of images from items (**c**) and (**d**). **f** The gradient obtained for the image from item (**b**)

**Fig. 5:**
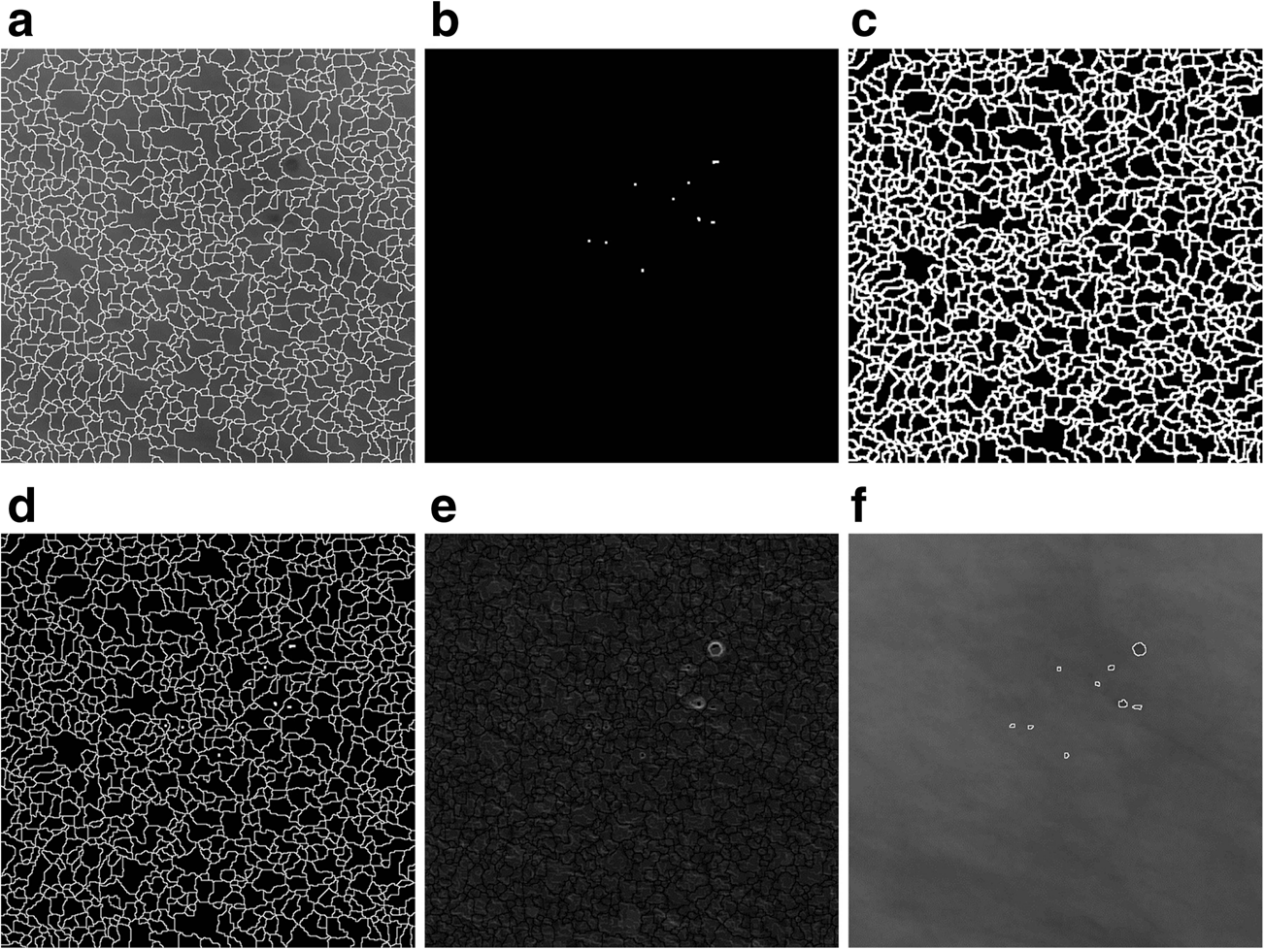
An illustration of extracting the microcalcifications shape. Part 2. **a** The result of the watershed segmentation, i.e., the external marker that has been overlaid on the image from Fig. 4b. **b** The internal marker. **c** The dilation of the external marker from item (**a**). **d** The logical sum of the external and internal markers. **e** The result of the minima imposition operation for the gradient image from Fig. 4f and the complete marker from Fig. 5d. **f** Output watershed lines overlaid on the image from Fig. 4b

The next step in working with mammograms is to extract the microcalcification shape. The methods discussed in the previous subsection were a way of determining the masks of microcalcifications, but their shape is, in the majority of cases, dependent on the morphological operations executed, such as the *emax* and the reconstruction [26]. The watershed segmentation [26] coupled with the use of the so-called markers which prepared the image for segmenting and control this process was used to find the shape of microcalcifications. Using markers provides additional knowledge about the objects for the segmentation process and makes their extraction more efficient. It also reduces oversegmentation. A marker is defined as a cohesive area of pixels belonging to the image. An internal marker is related to the object that should be extracted, while an external marker is related to the background. Further down, the sets of internal markers and external markers will be referred to, respectively, the internal marker and the external marker, and the sum of these two sets—simply as a marker. The next steps in segmenting the shape of microcalcifications are as follows:

1. The first is to find the mask of regional minima (assuming an 8-neighborhood in the analyzed image) for the mammogram image presented in Fig. 4b filtered through the C-O operation and then inverted. The image of regional minima generated at this step contains areas of microcalcifications as well as other artifacts in the image (Fig. 4d).
2. Having obtained an image of a regional minima mask, its intersection with the map of microcalcifications generated by the morphological microcalcification detection presented in Fig. 4c should be found. The result of this intersection will constitute an internal marker (Fig. 4e).
3. Then, the morphological gradient of the filtered and inverted input image referred to an item 1 is calculated. The definition of the morphological gradient used for image *I* is the result of subtracting the dilation *δ* and erosion *ε* with the use of structural element: 3 × 3 pixels in size.
  3 $$ gra{d}_B(I)={\delta}_B(I)-{\varepsilon}_B(I) $$
  The result is presented in Fig. 4f, whereas the image of the gradient has been multiplied times 10 to better distinguish details.
4. The inverted and filtered image from Fig. 4b should be subjected to the watershed segmentation in order to obtain an external marker which will consist of the output watershed lines. In Fig. 5a, the marker lines have been overlaid on the image from Fig. 4b.
5. The pixels of the internal marker must not touch the pixels of the external one. For this purpose, the image generated by dilating the external marker image should be subtracted from the image of the internal marker from Fig. 4e. The dilation operation on the external marker is presented in Fig. 5c—the lines from the marker image have been widened. The new internal marker should be added to the external marker (not widened) to obtain the complete marker image (Fig. 5d).
6. At the next computational step, the gradient undergoes the *minima imposition* operation [36] in which the argument is the complete marker obtained in item 5. This operation means that the only regional minima that remain in the gradient will be found in the places “marked” by the marker. The result of the minima imposition operation is presented in Fig 5e, whereas the output image has been multiplied times 10 to better distinguish details. The watershed segmentation is then carried out on a gradient thus modified. Its results consist in watershed lines running along the contour of microcalcifications, without the oversegmentation effect. The result of this method for the image from Fig. 3a is presented in Fig. 5f—the watershed lines have been overlaid on the inverted image from Fig. 4b in order to better present the areas of microcalcifications identified by them.

### Methods of Measuring and Assessing Microcalcification Segmentations Carried Out

The accuracy of microcalcification segmentation in mammograms from the DDSM database was estimated by measuring four indices, namely the similarity index $SI=\frac{2\cdot \left|M\cap E\right|}{\left|M\right|+\left|E\right|}$, the overlap fraction $OF=\frac{\left|M\cap E\right|}{\left|E\right|}$, the overlap value $OV=\frac{\left|M\cap E\right|}{\left|M\cup E\right|}$, and the extra fraction $OF=\frac{\left|M\cap \overline{E}\right|}{\left|E\right|}$.

Where

- *M* denotes regions of microcalcifications identified by the computer method, while |*M*| is the number of pixels.
- *E* represents areas of microcalcifications traced by the expert—a breast radiologist, while |*E*| is the number of pixels in the traced regions.
- |*M* ∩ *E*|, |*M* ∪ *E*| represent, respectively, the number of pixels in the common area and the number of all pixels in the *M* and *E* regions.

Using the four indices—SI, OF, OV, and EF—makes it possible to exhaustively compare the similarity and differences between the analyzed regions M and R and determine the overlap fraction, the underestimation, and the extra fraction. In [8, 9], only the OV index was analyzed. During research work, ROIs with the constant dimensions of 512 × 512 pixels were analyzed, while for mammograms from the DDSM database, there are GTA contours identifying the areas in which microcalcifications occur. Therefore, a radiologist participated in the experiments carried out and made the appropriate assessments of the detected or undetected actual and assumed microcalcification signals, namely

- If the computer method identified a microcalcification in the GTA area correctly, it was classified as true positive (TP).
- If the computer method did not identify a microcalcification in the GTA area correctly (the microcalcification does not occur in the area marked), it was classified as false positive (FP).
- If the computer method did not indicate a microcalcification in the GTA area even though it was there, this represented a false-negative case (FN).
- If the computer method produced a microcalcification signal outside of the GTA area, this represented a case of FP.

A radiologist’s assessment was used to calculate the mean sensitivity (4) depending on the number of false-positive signals per image (FPI).

4 $$ Sensitivity=T{P}_S/\left(T{P}_S+F{N}_S\right) $$

In contrast, the sensitivity was not analyzed in [8, 9]. In [8, 9], the segmentation was performed for every microcalcification separately and in addition for patches of various sizes, but after the previous initiation of the active contour, which can, unfortunately, be a painstaking and time-consuming activity if the number of microcalcifications is large.

### Selecting Parameters in Computer Method

Table 1 presents the values of parameters established for the morphological detection of microcalcifications using 20 pre-selected ROIs from mammograms from the DDSM database, namely 10 benign and 10 malignant cases The 20-element training set included ROIs containing microcalcifications of various shapes, sizes, numbers, and distribution as well as brightness levels. The watershed segmentation carried out after the microcalcifications are detected is automated and requires no parameters to be used. The parameters presented in Table 1 are selected so that the SI, OF, and OV indices are the highest possible, while the EF index is as low as possible.

**Table 1:** The established values of parameters of the morphological detection and extraction of microcalcifications

Parameter	diff	Th	h	nPxls	minPxls	maxPxls
Value	21	4	5	50	10	70

*diff* the value used in the gray level difference at stage 1; *Th* the value of the threshold at stage 2; *h* the height in the extended maximum (2); *nPxls* the maximum number of pixels of the detected area of microcalcification: vertically, horizontally, or transversely left or right; *minPxls* the minimal number of pixels in the area of the microcalcification being detected; *maxPxls* the maximal number of pixels in the area of the microcalcification being detected

## Results and Discussion

**Fig. 6:**
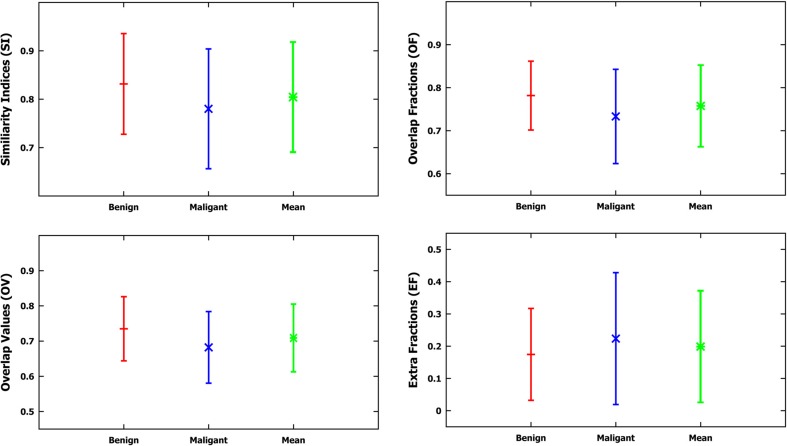
Graphs of the mean value and the standard deviation based on measurements of four indices: SI, OF, OV, and EF for the applied method, compared to the contours drawn by the radiologist based on the data from Table 2

After the parameters necessary to control the segmentation had been established, the method of detecting and segmenting microcalcifications was tested on the remaining 200 mammograms, using segmentations done manually by a radiologist and GTA contours. The results of these segmentations are presented in Table 2. Table 2 presents calculated statistical parameters such as the maximum value (max), minimum value (min), the mean value (mean), and the standard deviation (SD) of the following calculated indices: SI, OF, OV, and EF. Figure 6 shows a graph of data from Table 2. Table 3, in turn, presents

- The number of ROIs depending on the false positive per image (FPI) examples obtained (there are altogether 200 ROIs).
- The mean sensitivity values of detected microcalcifications depending on the detected false-positive (FP) examples.
- Standard deviations (SD), minimum values (min), and maximum values (max).

**Table 2:** Measurements (min, max, mean, SD—standard deviation) of indices: similarity (SI), overlap fraction (OF), overlap value (OV), and extra fraction (EF) for segmentations carried out on mammograms from the DDSM database, specifically 100 benign cases, 100 malignant cases, and jointly on all analyzed 200 ROIs

	SI	OF	OV	EF		SI	OF	OV	EF
Mean	0.831	0.781	0.735	0.174	Mean	0.780	0.733	0.682	0.223
SD	0.104	0.080	0.091	0.142	SD	0.123	0.109	0.101	0.204
Min	0.531	0.482	0.395	0.018	Min	0.432	0.415	0.341	0.021
Max	0.952	0.902	0.842	0.583	Max	0.924	0.865	0.825	0.653
Benign: computer method versus expert					Malignant: computer method versus expert				
	SI	OF	OV	EF					
Mean	0.805	0.757	0.708	0.198					
SD	0.113	0.094	0.096	0.173					
Min	0.432	0.415	0.341	0.018					
Max	0.952	0.902	0.842	0.653					
Benign and malignant: computer method versus expert

**Table 3:** Experiment results for the test set based on 200 mammograms from the DDSM database

FPI	0	1	2	3	4	5	6	7	8
Number of ROIs	24	36	32	34	20	8	12	22	12
Mean sensitivity	0.781	0.793	0.813	0.818	0.808	0.806	0.804	0.798	0.776
SD	0.116	0.154	0.143	0.153	0.108	0.04	0.042	0.052	0.062
Min	0.5	0.5	0.5	0.5	0.5	0.666	0.666	0.5	0.5
Max	1	1	1	1	1	0.85	0.857	0.857	0.857

The first row shows the number of ROIs depending on the number of false positives per image (FPI), and the subsequent rows their corresponding mean values of sensitivity, standard deviations (SD), minimum values (min), and maximum values (max)

**Fig. 7:**
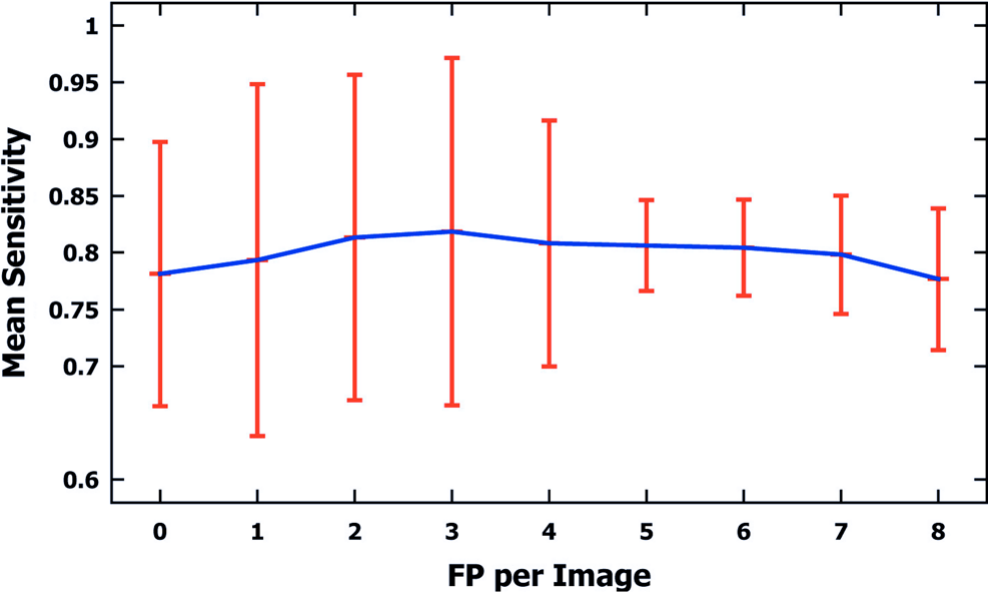
A graph of mean sensitivity values and standard deviations for microcalcifications detected in 200 mammograms from the DDSM database (100 malignant and 100 benign cases), depending on false-positive (FP) examples per image detected. Data based on Table 3

Figure 7 shows a graph of the data from Table 3.

Table 4 shows the time measurements, in seconds, of the morphological extraction and detection method for microcalcifications (M) used for the 200 analyzed mammograms. The presented method was implemented in the Matlab R2015a environment. Time was measured for a PC with an Intel Core i7 2 GHz processor. The average time for a single ROI 512 × 512 pixels in size amounts to 0.83 s, and this includes all steps of the method presented in this publication.

**Table 4:** Time measurements (min, max, mean, SD—standard deviation) of individual steps of the applied method (M), i.e., microcalcification detection (I) and extraction (II), expressed in seconds for 200 analyzed ROIs, 512 *×* 512 pixels in size, based on mammograms from the DDSM database

	I	II	M
Mean	0.514	0.321	0.836
SD	0.04	0.021	0.051
Min	0.420	0.312	0.730
Max	0.552	0.399	0.951

**Fig. 8:**
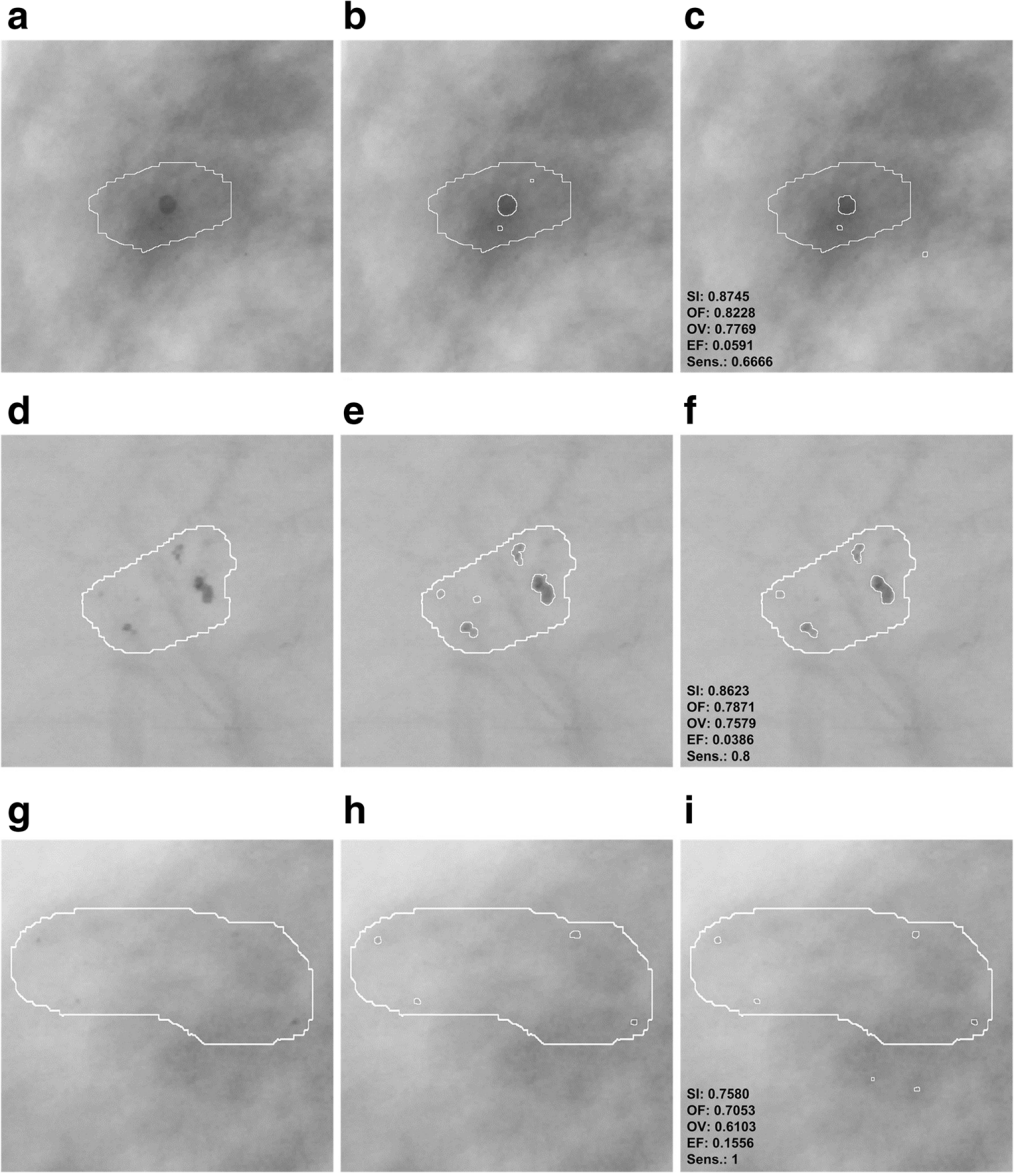
Benign cases—example results of microcalcification segmentation for selected mammograms from then DDSM database, together with the GTA contours marked. The images have been inverted in the gray scale to better bring out individual microcalcifications. *First column*: the GTA contour marked. *Second column*: contours of individual microcalcifications traced by a radiologist. *Third column*: results of segmenting microcalcifications using the computer method, with the calculated indices. **a**–**c** Image A_1480_1.LEFT_MLO. **d**–**f** A_1551_1.LEFT_MLO. **g**–**i** A_1553_1.LEFT_MLO

**Fig. 9:**
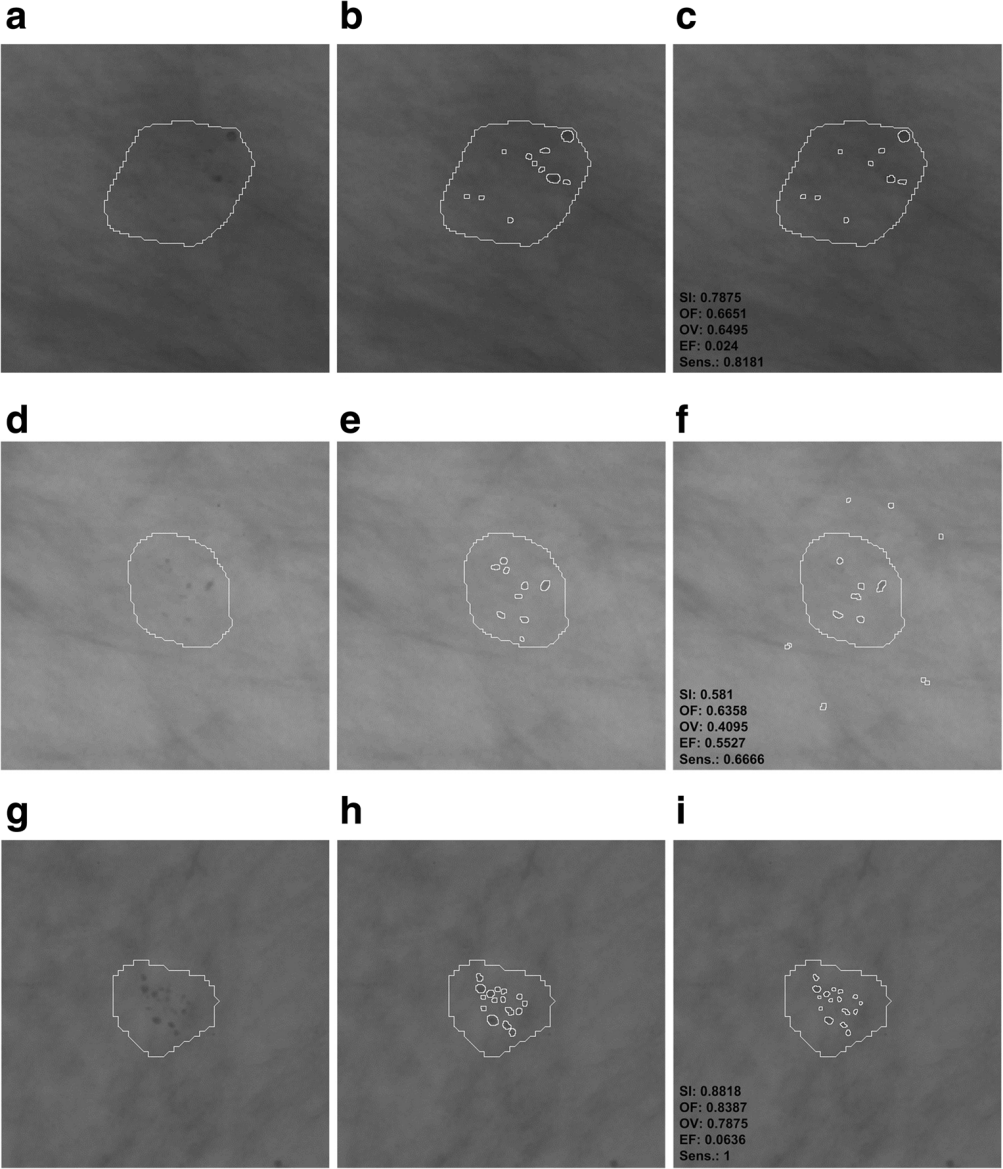
Malignant cases—example results of microcalcification segmentation for selected mammograms from the DDSM database, together with the GTA contours marked. The images have been inverted in the gray scale to better bring out individual microcalcifications. *First column*: the GTA contour marked. *Second column*: contours of individual microcalcifications traced by a radiologist. *Third column*: results of segmenting microcalcifications using the computer method, with the calculated indices. **a**–**c** Image A_1131_1.RIGHT_MLO. **d**–**f** A_1201_1.RIGHT MLO. **g**–**i** A_1214_1.LEFT MLO

Examples of differences in the segmentation of microcalcifications by the computer method presented in this publication and the contours manually traced by a radiologist are presented in Figs. 8 and 9. These are typical results obtained during the experiments carried out. In order to make microcalcification imaging easier, all examples of mammograms have been filtered according to stage 1 of the presented method and their gray levels have been inverted. In all examples from Figs. 8 and 9, GTA contours are superposed. Figure 8 shows example results for benign cases and Fig. 9 for malignant ones. The values of calculated indices are presented next to each example extracted by the watershed segmentation. Table 5 collates the results produced by active contour methods: MAC [8] and GAC [9] with those produced in this research work.

**Table 5:** A comparison of the results of two active contour methods: (MAC) [8] and (GAC) [9] with the method presented in this study, based on mammograms from the DDSM database [31, 32]

	MAC	GAC	M
Number of ROIs	128	1000	200
Size of ROI in pixels	81 *×* 81	From 20 *×* 20 to 41 *×* 41	512 *×* 512
Mean OV: benign cases	–	0.55	0.735
Mean OV: malignant cases	–	0.49	0.682
Mean OV: malignant and benign cases	0.61	0.52	0.708
Mean time in seconds for a single ROI	–	–	0.836
Mean time in seconds for a single microcalcification	0.42	0.4	–

The table compares the number of ROIs analyzed, their size in pixels, the mean values of the overlap value (OV) for benign and malignant cases, as well as for the benign and malignant cases together. The table also presents the mean time of executing the method in accordance with the adopted segmentation approach

The experiments completed and the research results presented in Tables 2, 3, 4, and 5 and Figs. 6, 7, 8, and 9 justify the following statements:

- In all experiments done on 200 ROIs 512 × 512 pixels in size, the average values of the SI, OF, OV, and EF indices amounted to, respectively, 80.5, 75.7, 70.8, and 19.8%. Higher values of SI, OF, and OV indices and a lower value of the EF index were obtained for benign cases, which are relatively larger and less numerous than malignant ones. The values of the SI, OF, OV, and EF indices for 100 analyzed ROIs containing benign lesions are 83, 78, 73.5, and 14%, while for those with malignant lesions they equal 78, 73, 68, and 22%. In [9], only the OV index was analyzed, and just as here, higher values were obtained for benign lesions than for malignant ones. In [8], in turn, only the sizes of microcalcifications were distinguished, and no results of experiments for types of microcalcifications are presented. This study produced higher average values of the OV index than in [9] (52%) and in [8] (61%). However, it is worth noting that Duarte et al. [9] researched 1000 ROIs from mammograms from the DDSM database, so significantly more experiments were carried out than in this publication (altogether 220 ROIs, with 20 used to determine the parameters of the method, and tests carried out on the remaining 200). What is more, in [9], the researchers analyzed various types of microcalcifications and for different types of breast tissues according to their classification to four tissue density categories [37]. In this study, two types of microcalcifications were analyzed, namely those which are symptoms of malignant cases and those which represent benign cases, and they are generally fatty breast cases. Unfortunately, as the manual tracing of individual microcalcifications can be very time consuming for the expert (as long as 30 min for a single ROI), this forms an obstacle to conducting a large number of experiments and significantly prolongs their time. In [8], 128 clusters of microcalcifications were analyzed.
- According to the data from Table 3, there were eight supposed microcalcifications at the most. In the experiments completed, the most frequent ROIs had one, two, and three supposed microcalcifications, and for these cases the standard deviations are the greatest. The minimal number of microcalcifications that occurred was 5 and the lowest value of the standard deviation can be observed for this group. The mean sensitivity in all the experiments amounted to 80% and reached the maximum value of 81% for FPI 2 and 3. On the contrary, the lowest value of 77% occurred when the maximum number of FPI signals was equal to 8. The most frequent cases which reduce the mean sensitivity of the method are those where few microcalcifications occur in the mammogram but are not all detectable by the computer method. For example, there are three microcalcifications in Fig. 8b, but the computer method missed one microcalcification—Fig. 8c, so its sensitivity amounts to only 0.66. This relationship becomes vague when there are more microcalcifications, and then omitting a few of them does not significantly impact the sensitivity values obtained. The appropriate examples are illustrated by the following pairs of Fig. 8e and f as well as Fig. 9b and c. Figure 8e contains five microcalcifications, so not detecting one of them yields the sensitivity of 0.8—Fig. 8f. In the example from Fig. 9b, the radiologist has found 11 microcalcifications, and if the computer method misses two, the sensitivity amounts to 0.81. Figure 9e, in turn, contains nine microcalcifications, so if the computer detection misses three, this represents the sensitivity of 0.66. According to the data from Table 3, sensitivity falls to 0.5 in the worst case and is equal to 1 in the best case, which means that all microcalcifications had been found.
- The average completion time of all steps of the computer method for ROIs 512 × 512 pixels in size amounted to 0.83 s and consisted of 0.51 s for the morphological detection of microcalcification and 0.32 s for the watershed segmentation. In comparison, the authors of [8] analyzed ROIs 81 × 81 pixels in size and reported that the average segmentation time of a single microcalcification was 0.42 s. Duarte et al. [9] also give the average segmentation time for a single microcalcification, which amounted to 0.4 s for analyzed ROIs whose dimensions ranged from 20 × 20 to 41 × 41 pixels. It should be noted that the active contour methods presented in [8, 9] require a manual initialization for every single microcalcification, which represents a significant limitation because it prolongs the segmentation process, particularly if a large number of ROIs is analyzed and they contain an even greater number of microcalcifications. In summary, the solutions proposed in this publication are more practical because they do not require initializing in every instance—they allow the segmentation process to be automated not just for single microcalcifications but for many at the same time, inside ROIs larger in size and within a shorter time; and they do not require initializing in every instance—they allow the segmentation process to be automated not just for single microcalcifications but for many at the same time, inside ROIs larger in size and within a shorter time.

## Summary and Conclusion

This publication presents a computer method for detecting and segmenting microcalcifications in mammograms from the DDSM database. It uses morphological transformations and is composed of two parts. The first part detects microcalcifications morphologically, thus allowing the approximate area of their occurrence to be determined, the contrast to be improved, and noise to be reduced in the mammograms. Then, the watershed segmentation of microcalcifications is performed. In the experiments carried out for 200 ROIs taken from mammograms from the DDSM database, the measured values of the SI, OF, OV, and EF indices amounted to, respectively, 80.5, 75.7, 70.8, and 19.8%. Higher values of the SI, OF, and OV indices and a lower value of the EF index were obtained for benign cases than for malignant ones. Compared to other solutions presented in [8, 9], the process of microcalcification segmentation was automated and the computer methods used achieved at a significant speed. In the experiments completed, the average running time of the entire processing of a single ROI 512 × 512 in size amounted to 0.83 s. Increasing the number of cases from the DDSM database, particularly to include different types of microcalcifications according to the classification presented in [37], should be considered in further research. The segmentation results produced by the computer method should be evaluated by two experienced breast radiologists, and this would additionally allow the consistency of these evaluations to be compared. It should be noted that the DDSM database is not new and it will be worthwhile to add examination results produced by the newest generation of mammographs. On the other hand, the publicly accessible DDSM database is the only one containing the highest number of images together with the detailed location of lesions and their descriptions, so many researchers are willing to use it.

## References

[1] Arodź T, Kurdziel M, Popiela TJ, Sevre EO, Yuen DA (2006). Detection of clustered microcalcifications in small field digital mammography. Comput Methods Prog Biomed.

[2] Andreadis II, Spyrou GM, Nikita KS (2015). A CAD scheme for mammography empowered with topological information from clustered microcalcifications atlases. IEEE J Biomed Health Inform.

[3] Elter M, Horsch A (2009). CADx of mammographic masses and clustered microcalcifications: a review. Med Phys.

[4] Nishikawa RM (2007). Current status and future directions of computer-aided diagnosis in mammography. Comput Med Imaging Graph.

[5] Paquerault S, Yarusso LM, Papaioannou J, Jiang Y, Nishikawa RM (2004). Radial gradient-based segmentation of mammographic microcalcifications: observer evaluation and effect on CAD performance. Med Phys.

[6] Sharma S, Khanna P (2015). Computer-aided diagnosis of malignant mammograms using Zernike moments and SVM. J Digit Imaging.

[7] Singh SP, Urooj S (2016). An improved CAD system for breast cancer diagnosis based on generalized pseudo-Zernike moment and Ada-Dewnn classifier. J Med Syst.

[8] Arikidis NS, Karahaliou A, Skiadopoulos S, Korfiatis P, Likaki E, Panayiotakis G, Costaridou L (2010). Size-adapted microcalcification segmentation in mammography utilizing scale-space signatures. Comput Med Imaging Graph.

[9] Duarte MA, Alvarenga AV, Azevedo CM, Calas MJG, Infantosi AF, Pereira WC (2015). Evaluating geodesic active contours in microcalcifications segmentation on mammograms. Comput Methods Prog Biomed.

[10] Mohanalin J, Kalra PK, Kumar N (2009). Microcalcification segmentation using normalized Tsallis entropy: an automatic q calculation by exploiting type II fuzzy sets. IETE J Res.

[11] Oliver A (2012). Automatic microcalcification and cluster detection for digital and digitized mammograms. Knowl-Based Syst.

[12] Andreadis I, Spyrou G, Nikita K (2011). A comparative study of image features for classification of breast microcalcifications. Meas Sci Technol.

[13] He W, Hogg P, Juette A, Denton ER, Zwiggelaar R (2015). Breast image pre-processing for mammographic tissue segmentation. Comput Biol Med.

[14] Mete M, Sirakov NM (2012). Dermoscopic diagnosis of melanoma in a 4D space constructed by active contour extracted features. Comput Med Imaging Graph.

[15] Szczypiński PM, Strzelecki M, Materka A, Klepaczko A (2009). Mazdaa software package for image texture analysis. Comput Methods Prog Biomed.

[16] Diaz-Huerta CC, Felipe-Riveron EM, Montaño-Zetina LM (2014). Quantitative analysis of morphological techniques for automatic classification of micro-calcifications in digitized mammograms. Expert Syst Appl.

[17] Tsai DY (2004). Medical image classification using genetic-algorithm based fuzzy-logic approach. J Electron Imaging.

[18] Wei L, Yang Y, Nishikawa RM (2009). Microcalcification classification assisted by content-based image retrieval for breast cancer diagnosis. Pattern Recogn.

[19] Ren J (2012). ANN vs. SVM: which one performs better in classification of MCCS in mammogram imaging. Knowl-Based Syst.

[20] Betal D, Roberts N, Whitehouse GH (1997). Segmentation and numerical analysis of microcalcifications on mammograms using mathematical morphology. Br J Radiol.

[21] Halkiotis S, Botsis T, Rangoussi M (2007). Automatic detection of clustered microcalcifications in digital mammograms using mathematical morphology and neural networks. Signal Process.

[22] Nieniewski M: Watershed extraction of the exact shape of microcalcifications in mammograms. In: Computer Recognition Systems. Springer, 2005, pp 635–643

[23] Xu S, Liu H, Song E (2011). Marker-controlled watershed for lesion segmentation in mammograms. J Digit Imaging.

[24] Chen Z, Strange H, Oliver A, Denton ER, Boggis C, Zwiggelaar R (2015). Topological modeling and classification of mammographic microcalcification clusters. IEEE Trans Biomed Eng.

[25] Batchelder KA, Tanenbaum AB, Albert S, Guimond L, Kestener P, Arneodo A, Khalil A (2014). Wavelet-based 3D reconstruction of microcalcification clusters from two mammographic views: new evidence that fractal tumors are malignant and Euclidean tumors are benign. PLoS ONE.

[26] Soille P: Morphological image analysis: principles and applications. Springer Science & Business Media, 2013

[27] Nieniewski M: Digital image segmentation: watershed segmentation methods. Academic Publishing House Exit, 2005

[28] Roerdink JB, Meijster A (2000). The watershed transform: definitions, algorithms and parallelization strategies. Fundam Informaticae.

[29] Vincent L, Soille P: Watersheds in digital spaces: an efficient algorithm based on immersion simulations. IEEE Trans Pattern Anal Mach Intell (6): 583–598, 1991

[30] Nieniewski M (2004). Extraction of diffuse objects from images by means of watershed and region merging: example of solar images. IEEE Trans Syst Man Cybern B Cybern.

[31] Heath M, Bowyer K, Kopans D, Kegelmeyer Jr P, Moore R., Chang, K, Munishkumaran S: Current status of the digital database for screening mammography. In: Digital mammography. Springer, 1998, pp 457–460

[32] Heath M, Bowyer K, Kopans D, Moore R, Kegelmeyer WP: The digital database for screening mammography. In: Proceedings of the 5th International Workshop on Digital Mammography, 2000, pp 212–218

[33] Agrawal P, Vatsa M, Singh R (2014). Saliency based mass detection from screening mammograms. Signal Process.

[34] Jen CC, Yu SS (2015). Automatic detection of abnormal mammograms in mammographic images. Expert Syst Appl.

[35] Cheng HD, Cai X, Chen X, Hu L, Lou X (2003). Computer aided detection and classification of microcalcifications in mammograms: a survey. Pattern Recogn.

[36] Gonzalez RC, Woods RE: Digital image processing, 3rd edition. Prentice Hall, 2007

[37] Balleyguier C, Ayadi S, Van Nguyen K, Vanel D, Dromain C, Sigal R (2007). Birads classification in mammography. Eur J Radiol.

